# In-Depth Exploration of the Coloration Mechanism of *Iris dichotoma* Pall. via Transcriptomic and Metabolomic Analyses

**DOI:** 10.3390/plants14091387

**Published:** 2025-05-04

**Authors:** Yalin Yu, Xiaojing Qiang, Fan Huang, Xiuzheng Huang, Lei Liu

**Affiliations:** Institute of Grassland Research, Chinese Academy of Agricultural Sciences, Hohhot 100081, China; yuyalin2025@163.com (Y.Y.); qiangxiaojing11@163.com (X.Q.); huangfan@caas.cn (F.H.); 19917621903@163.com (X.H.)

**Keywords:** *Iris dichotoma* Pall., transcriptome, metabolome, anthocyanins, delphinidin

## Abstract

*Iris dichotoma* Pall., renowned for its high ornamental value, is frequently cultivated in flowerbeds and courtyards, endowing garden landscapes with unique allure. Dark-hued flowers are widely regarded as more aesthetically appealing. This study utilized the petals of two distinct *Iris dichotoma* Pall. phenotypes as research materials to investigate the underlying mechanism of flower color formation. The purple-flowered *Iris dichotoma* Pall. was designated as Group P, and the white-flowered one as Group W. A comprehensive integrative analysis of the transcriptome and metabolome of the two petal types was carried out. Metabolomic analysis revealed that the contents of several anthocyanin derivatives, including delphinidin, petunidin, malvidin, peonidin, and procyanidin, were significantly higher in purple petals compared to white petals, with delphinidin exhibiting the highest content. The transcriptomic analysis detected 6731 differentially expressed genes (DEGs) between the white and purple petal types. Specifically, 3596 genes showed higher expression levels in purple petals, while 3135 genes exhibited lower expression levels in purple petals compared to white petals. Ten phenylalanine ammonia-lyase (PAL) genes, two chalcone synthase (CHS) genes, one anthocyanidin reductase (ANR) gene, one 4-coumarate-CoA ligase (4CL) gene, one dihydroflavonol 4-reductase (DFR) gene, one flavanone 3′-hydroxylase (F3′H) gene, and one flavonol synthase (FLS) gene were identified; they all had purple petals displaying higher expression levels than white petals. This research uncovers the potential formation mechanism of anthocyanins in the two *Iris dichotoma* Pall. types, thereby furnishing a theoretical foundation for floral breeding endeavors.

## 1. Introduction

*Iris dichotoma* Pall., a perennial herbaceous plant, exhibits unique value in ecological, horticultural, and medicinal fields due to its distinctive anthocyanin characteristics, making it suitable for landscape beautification. However, under natural conditions, its petals are predominantly limited to purple and white, which appear relatively monotonous in cultivated settings. Although there have been reports of new color variations in irises, the molecular mechanisms underlying petal pigmentation in *Iris dichotoma* remain poorly understood. Numerous plant species produce a narrow spectrum of flavonoids, which leads to a narrow variety of floral colors [1]. The current paucity of data on petal pigment composition and the molecular regulatory networks governing floral coloration represents a critical bottleneck in ornamental plant breeding programs. Over the past few decades, notable breakthroughs have been achieved in deciphering the transcriptional and post-translational regulatory networks underlying anthocyanin accumulation in representative plant systems [2,3,4]. However, the molecular mechanisms in non-model organisms, including *Iris dichotoma*, still require further exploration. The presence of genes regulating flavonoid biosynthesis allows for the modification of flower colors through the overexpression of heterologous genes or the suppression of endogenous genes. Transgenic roses and carnations that overexpress the genes for flavonoid 3′,5′-hydroxylase, for example, accumulate delphinidin and create unique blue flowers that are now on the market [5,6,7]. Similarly, transgenic *Nierembergia* accumulating pelargonidin has been developed, resulting in flowers with a novel pink color [8]. Additionally, hybridization experiments between *Iris dichotoma* and *Belamcanda chinensis* have yielded offspring with more vibrant and diverse flower colors, such as the summer ornamental iris “Sweet Princess”, which exhibits enhanced aesthetic value [9]. Given that a broader spectrum of flower colors aligns more closely with public aesthetic preferences, it is of critical importance to investigate the anthocyanin biosynthesis pathways in *Iris dichotoma*. Such research will not only advance our understanding of the molecular mechanisms underlying flower pigmentation but also facilitate the development of novel cultivars with improved ornamental value.

The generation of petal colors entails a cascade of intricate biochemical reactions, with a multitude of factors contributing to this complex process. These determinants include anthocyanin concentration, gene expression levels, external environmental elements such as light intensity, temperature, and humidity, the plant’s ontogenetic stage, internal physiological factors including pH, and soluble sugar content. Anthocyanins play a pivotal role in bestowing plants with a diverse spectrum of hues in their floral and fruit structures [10,11,12,13]. Beyond enhancing the aesthetic appeal of plants, which can have a profound impact on human emotional well-being, anthocyanins are crucial for the facilitation of insect-mediated pollination and the dissemination of seeds, thereby serving as a linchpin in the reproductive success of plants [2,4]. As water-soluble pigments are ubiquitously distributed across the plant kingdom, anthocyanins are acknowledged as the primary chromophores responsible for plant coloration. Moreover, they endow plants with enhanced resilience. They confer the ability to counteract biotic challenges posed by viruses, bacteria, and other pathogens; shield plants from the deleterious effects of excessive visible and ultraviolet radiation; and effectively detoxify the overproduction of reactive oxygen species under abiotic stress conditions [14]. From a human health perspective, anthocyanins exhibit a panoply of bioactive properties. Extensive research has demonstrated that anthocyanins, as potent natural antioxidants, can efficiently scavenge free radicals, thereby exerting remarkable anti-oxidative and anti-aging effects. Additionally, they have been implicated in modulating inflammatory responses, mitigating the risk of obesity, forestalling the onset of chronic metabolic disorders, safeguarding cardiovascular function, and promoting ocular health [15,16,17,18,19,20]. Structurally, anthocyanins exist in the form of glycosides, typically conjugating with one or more monosaccharides, including glucose, galactose, rhamnose, xylose, or arabinose [18]. Biochemically, anthocyanins can be taxonomically classified into six major groups: pelargonidin (Pg), cyanidin (Cy), delphinidin (Dp), peonidin (Pn), petunidin (Pt), and malvidin (Mv) [21,22,23,24,25].

The anthocyanin biosynthesis process is mostly governed by structural enzyme genes originating from the phenylpropanoid metabolic pathway, a crucial secondary metabolic pathway in numerous plants. The biosynthesis of anthocyanins begins with Phenylalanine. This precursor is successively catalyzed by Phenylalanine ammonia-lyase (PAL), Cinnamate 4-hydroxylase (C4H), and 4-coumarate-CoA ligase (4CL) to be converted into the key intermediate *p*-coumaroyl-CoA. Subsequently, *p*-coumaroyl-CoA is further transformed into yellow chalcone under the catalysis of Chalcone synthase (CHS). This step lays the basic carbon skeleton for the formation of flavonoids [26,27,28,29,30]. Next, Chalcone isomerase (CHI) and Flavanone 3-hydroxylase (F3H) act successively to convert chalcone into naringenin and dihydrokaempferol. At this point, the anthocyanin synthesis pathway branches into three directions: the F3′5′H, F3′H, and DFR branches. In the F3′5′H branch, the substrate dihydrokaempferol is catalyzed by F3′5′H to generate dihydromyricetin. Dihydromyricetin is further reduced to leucodelphinidin under the action of DFR (dihydroflavonol 4-reductase), and leucodelphinidin is converted into delphinidin under the catalysis of ANS. These pigments endow plants with blue, purple, and other colors. In the F3′H pathway, Flavonoid 3′-hydroxylase (F3′H) further catalyzes the formation of dihydroquercetin, which is an important precursor in anthocyanin synthesis. This sequence of metabolic reactions forms the fundamental stages of the anthocyanin biosynthesis pathway, guaranteeing the consistent production of anthocyanins in plants. Finally, these colorless dihydroflavonols are reduced to leucoanthocyanidins by Dihydroflavonol 4-reductase (DFR). Then, anthocyanidin synthase (ANS) catalyzes the formation of colored anthocyanins. Subsequently, UDP-glucose: flavonoid 3-*O*-glucosyltransferase (UFGT) catalyzes the conversion of unstable anthocyanins into stable anthocyanins. With the assistance of Glutathione-*S*-transferase (GST), the stable anthocyanins are transported from the cytoplasm to the vacuole for storage. In the DFR branch, without the participation of F3′5′H and F3′H, DFR directly acts on dihydrokaempferol, reducing it to leucoanthocyanidin. Under the catalysis of ANS, pelargonidin is generated, which usually makes plants appear orange-red.

**Figure 1:**
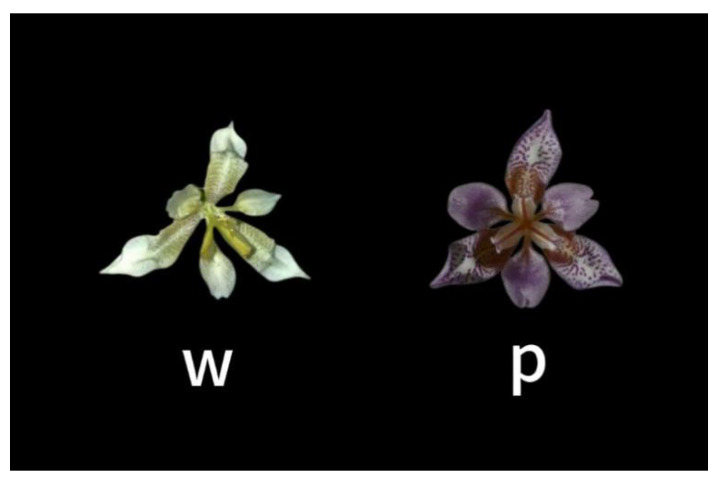
The phenotypes exhibit distinct flower colors: *Iris dichotoma* with (W) white flowers and (P) purple blooms.

This study investigated the metabolomes and transcriptomes of the petals of purple-flowered irises (P) and white-flowered irises (W) as referenced above (Figure 1). We conducted a comparative analysis of the metabolomes and transcriptomes to investigate the anthocyanin production process, anthocyanin concentration, and the transcription factors (TFs) associated with floral color development.

## 2. Results

### 2.1. Determination of Targeted Anthocyanin Metabolites in Flowers of Different Colors

**Figure 2:**
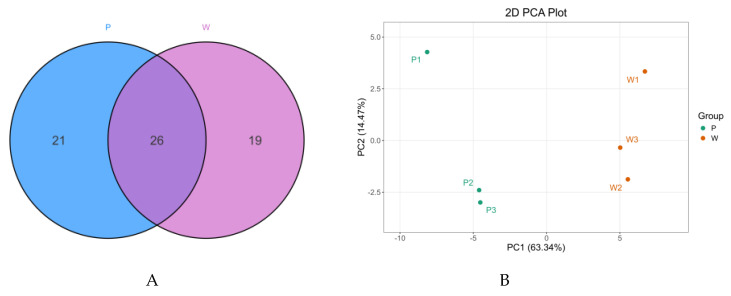

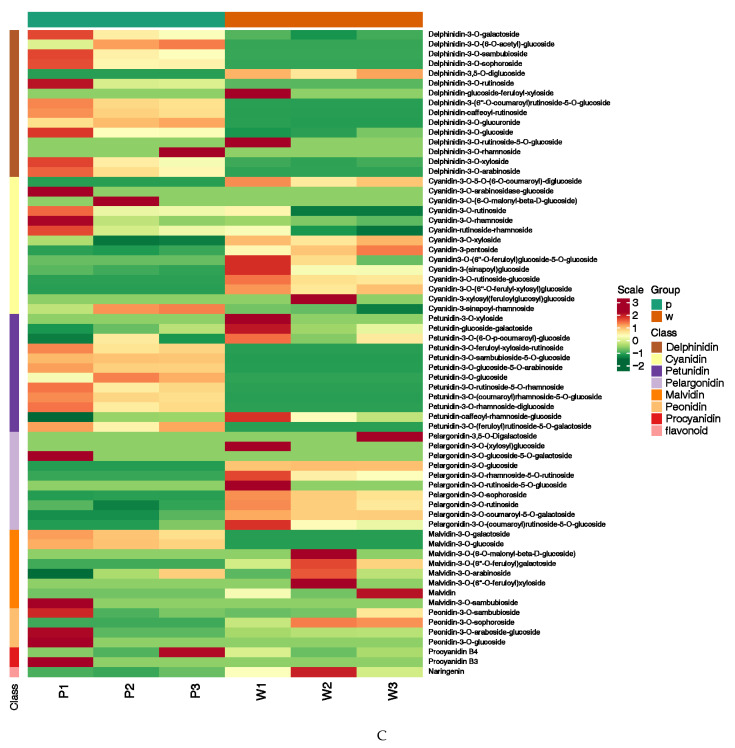
(**A**) A Venn diagram was constructed to illustrate the overlapping and unique features between the two comparison sets. (**B**) The metabolome data were put through principal component analysis (PCA). A PCA map has an *x*-axis that shows principal component 1 (PC1) and a *y*-axis that shows principal component 2 (PC2). (**C**) An analysis of differentially accumulated anthocyanins across the three experimental groups was carried out.

For the purpose of investigating the petals of two-colored iris flowers, we created a high-capacity metabolite library using UPLC/MS. Principal Component Analysis (PCA) was carried out (Figure 2A). The contribution rate of PC1 was 63.34% and that of PC2 was 14.74%. There was a significant separation, indicating an obvious differential trend between the two groups of data.

Anthocyanin-targeted metabolism identified 44 differentially accumulated DEMs (Differentially Expressed Metabolites), which could be classified into 8 major categories, namely petunidin (10), delphinidin (11), flavonoid (1), malvidin (4), peonidin (1), cyanidin (11), and pelargonidin (6). There were 26 of the same anthocyanins in purple and white flowers (Figure 2B). Approximately 24 DEMs showed increased abundance in purple petals, while 20 showed decreased abundance. The proportion of highly abundant petunidin and delphinidin was significantly higher in purple petals than that of low-abundant counterparts in white petals, suggesting their key roles in petal coloration.

The top six anthocyanin derivatives with the highest content in purple flowers all belonged to delphinidin. The content of these derivatives was significantly higher than that of delphinidin in white flowers. They included delphinidin-3-*O*-galactoside (7.08 μg/g), delphinidin-3-*O*-rutinoside (43.59 μg/g), delphinidin-3-(6″-*O*-coumaroyl) rutinoside-5-*O*-glucoside (122.88 μg/g), delphinidin-caffeoyl-rutinoside (26.16 μg/g), delphinidin-3-*O*-glucuronide (26.25 μg/g), and delphinidin-3-*O*-glucoside (7.02 μg/g). Evidently, delphinidin is essential for controlling how purple flowers develop their coloration.

**Figure 3:**
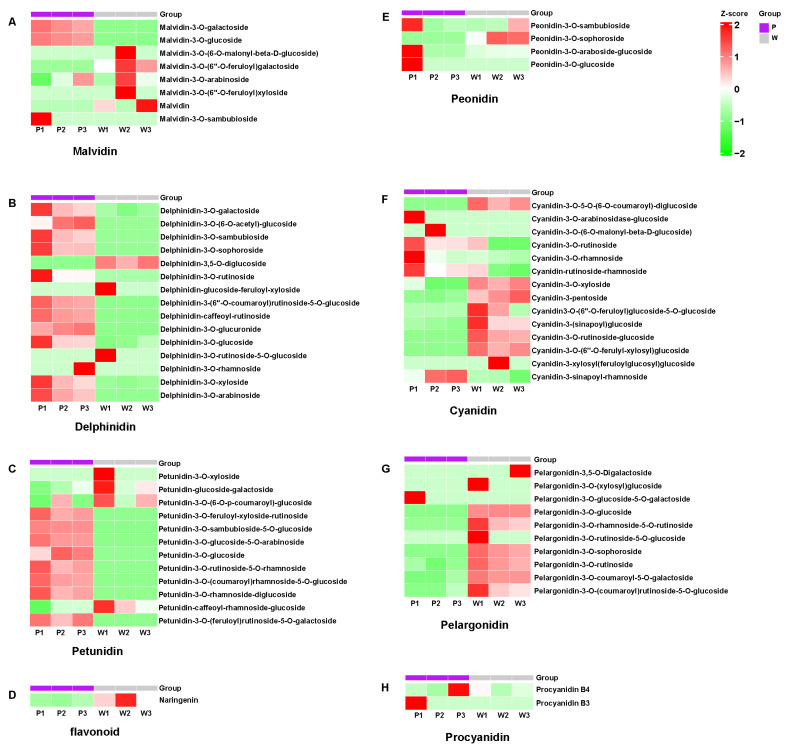
DAE Heatmap of Six Anthocyanins in Purple and White Petals. P1, P2, P3, W1, W2, and W3 represent three replicates of purple flowers and white flowers, respectively. Malvidin (**A**), Delphinidin (**B**), Petunidin (**C**), flavonoid (**D**), Peonidin (**E**), Cyanidin (**F**), Pelargonidin (**G**), and Procyannidin (**H**) in Purple Petals vs White Petals.

In this study, we constructed a Deep Autoencoder (DAE) heatmap to delve deeper into the differences in anthocyanins between purple and white flowers (Figure 3 and Figure S2). Leveraging the powerful feature extraction and dimensionality reduction capabilities of the DAE, we processed the acquired anthocyanin-related data to generate an intuitive and information-rich heatmap.

In the heatmap, the intensity of color directly reflects the relative content changes of anthocyanins in purple and white flowers. Judging from the overall color distribution, the purple-flower region exhibits significantly higher color intensity than the white-flower region in some key anthocyanin components, indicating a higher accumulation level of these anthocyanins in purple flowers. We found that 19 anthocyanins, including malvidin (malvidin-3-*O*-galactoside, malvidin-3-*O*-glucoside) (Figure 3A), delphinidin (delphinidin-3-(6″-*O*-coumaroyl) rutinoside-5-*O*-glucoside, delphinidin-caffeoyl-rutinoside, delphinidin-3-*O*-galactoside, delphinidin-3-*O*-xyloside, delphinidin-3-*O*-sambubioside, delphinidin-3-*O*-arabinoside, delphinidin-3-*O*-sophoroside, delphinidin-3-*O*-glucoside) (Figure 3B), petunidin (petunidin-3-*O*-rutinoside-5-*O*-rhamnoside, petunidin-3-*O*-rhamnoside-diglucoside, petunidin-3-*O*-feruloyl-xyloside-rutinoside, petunidin-3-*O*-(feruloyl) rutinoside-5-*O*-galactoside, petunidin-3-*O*-(coumaroyl) rhamnoside-5-*O*-glucoside, petunidin-3-*O*-glucoside-5-*O*-arabinoside, petunidin-3-*O*-sambubioside-5-*O*-glucoside) (Figure 3C), and cyanidin (cyanidin-3-*O*-rutinoside, cyanidin-rutinoside-rhamnoside) (Figure 3F), accumulate more in purple flowers than in white flowers. This suggests that these types of anthocyanins may regulate the accumulation and synthesis of anthocyanins. Through the construction and analysis of the DAE heatmap, we clearly revealed the differences in anthocyanin composition and content between purple and white flowers. This not only provides strong visual evidence for a deeper understanding of the flower-color formation mechanism but also lays a solid foundation for further research on the functions of anthocyanins in plant growth, development, and environmental adaptation.

### 2.2. Transcriptome Analysis of Petals

**Figure 4:**
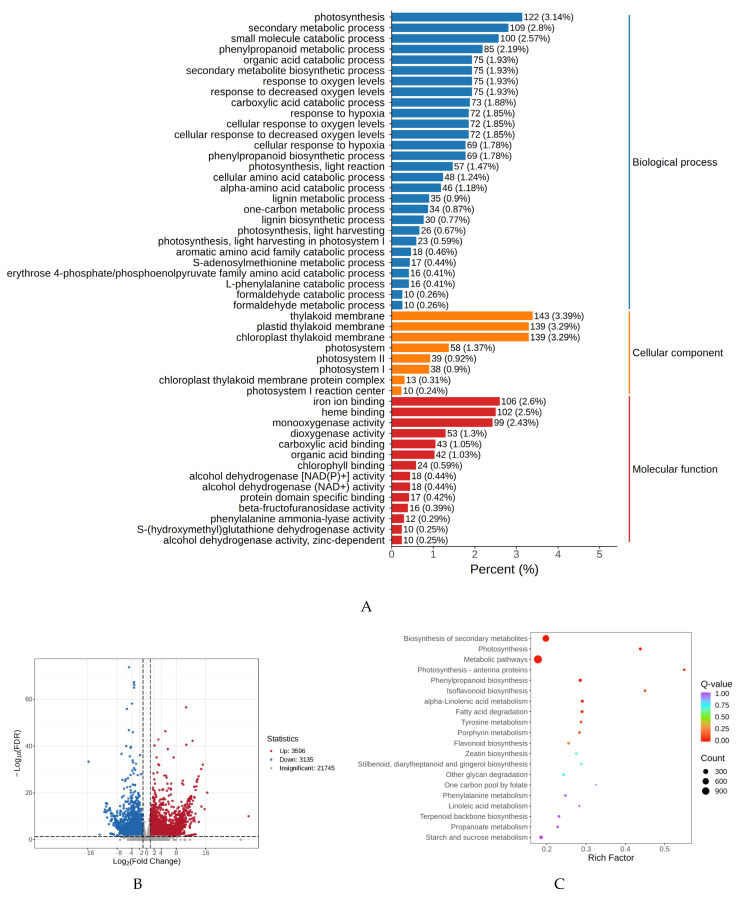
(**A**) GO classification in the P vs W. (**B**) Volcano map illustrating differentially expressed genes between two petal kinds exhibiting distinct colors. (**C**) KEGG classification in the P vs W.

The transcriptome analysis was conducted on six samples. In total, 46.1 GB of high-quality data were acquired, with each high-quality data sample attaining 6 GB. The proportion of Q30 bases was 91% or more. These transcriptome data indicate that the RNA-seq dataset is reliable for further research. All differentially expressed genes (DEGs) were mapped to the Gene Ontology (GO) database and then further categorized into three main categories: cellular components, molecular functions, and biological processes. (Figure 4A). In the cellular component category, most of the enrichments were observed in the “cellular process” and “metabolic process”. In the biological process category, the majority of DEGs were mapped to “cellular anatomical entity”. As for molecular functions, most DEGs were mapped to “binding” and “catalytic activity”. Regarding the Kyoto Encyclopedia of Genes and Genomes (KEGG) annotation results, 6731 DEGs from all samples were mapped to 138 KEGG pathways. The volcano plot analysis revealed that when comparing purple petals with white petals, a total of 6731 differentially expressed genes were identified. Among these genes, 3596 DEGs showed higher expression levels in purple petals, while 3135 DEGs exhibited lower expression levels in purple petals relative to white petals (Figure 4B). Many DEGs were enriched in pathways associated with anthocyanin accumulation, such as “Flavonoid biosynthesis” (Figure 4C).

To validate the credibility of the RNA-seq findings, 10 pivotal genes implicated in the anthocyanin biosynthesis pathway were chosen for quantitative real-time polymerase chain reaction (qRT-PCR). The relative expression magnitudes of these genes concurred with the RNA-seq outcomes, indicating the reliability of the RNA-seq data in this research.

### 2.3. Differential Expression of Anthocyanin Structural Genes

To delve into the accumulation of flavonoids in the leaves of *Iris dichotoma* in more detail, we investigated the gene expression patterns within the flavonoid pathway. Based on the transcriptome results, to gain a more profound comprehension of the fundamental mechanisms governing anthocyanin synthesis, we established the anthocyanin synthesis pathway. A total of 36 structural genes associated with anthocyanin biosynthesis were identified through screening, including PAL (12), CHS (10), ANR (5), 4CL (2), CHI (2), C4H (1), DFR (1), F3′H (1), and FLS (1). These genes demonstrated notable disparities in expression between purple-flowered and white-flowered plants. Specifically, the expression levels of 10 PAL genes (Cluster-21,093.0, log2FoldChange = 3.22; Cluster-24,453.0, log2FoldChange = 2.54; Cluster-3407.10, log2FoldChange = 1.62; Cluster-3407.14, log2FoldChange = 2.39; Cluster-3407.19, log2FoldChange = 2.48; Cluster-3407.5, log2FoldChange = 2.04; Cluster-3407.6, log2FoldChange = 1.77; Cluster-3407.7, log2FoldChange = 2.4; Cluster-3407.8, log2FoldChange = 2.28; Cluster-3407.9, log2FoldChange = 1.79), 2 CHS genes (Cluster-12,772.0, log2FoldChange = 2.57; Cluster-13,840.12, log2FoldChange = 2.95), 1 ANR gene (Cluster-7415.6, log2FoldChange = 3.74), 1 4CL gene (Cluster-12,604.0, log2FoldChange = 2.29), 1 DFR gene (Cluster-16,210.2, log2FoldChange = 9.67), 1 F3′H gene (Cluster-19,090.0, log2FoldChange = 2.67), and 1 FLS gene (Cluster-14,001.0, log2FoldChange = 2.46) were higher in purple-flowered plants compared to white-flowered ones.

### 2.4. Analysis of Key Transcription Factors Regulating Anthocyanin Synthesis

In the present study, through the comparison between purple petals and white petals, we identified 400 transcription factors (TFs) that displayed differential expression. Precisely, 153 TFs were found to have elevated expression levels in purple petals as contrasted with white petals, whereas 247 TFs showed reduced expression levels in purple petals when compared to white petals. These TFs included 22 WRKYs, 13 bHLHs, 18 MYBs, and several other types, all of which may be involved in anthocyanin biosynthesis. TFs with higher expression levels in purple-colored petals compared to white petals are likely to play a role in anthocyanin accumulation. We selected some TFs with an absolute log2FoldChange value greater than 2, including 15 WRKYs, 5 bHLHs, and 8 MYBs. Correlation analyses were then performed between these three types of TFs and the screened structural genes (Figure S3).

## 3. Discussion

The development of flower color is determined by certain places and temporal factors. Delphinidin is a plant pigment that causes plants to exhibit blue, magenta, and purple colors. It is the primary anthocyanin found in blackcurrants, eggplants, etc. [31,32,33]. In the present study, transcriptomic and metabolomic analyses were carried out on the purple and white petals of *Iris dichotoma*. The metabolomic analysis indicated that the contents of several anthocyanin derivatives related to delphinidin, petunidin, malvidin, peonidin, and procyanidin in purple flowers were significantly higher than those in white flowers, with delphinidin having the highest content. The transcriptomic analysis revealed that 6731 differentially expressed genes were detected in the two types of petals; 3596 genes exhibited significantly elevated expression levels in purple petals, whereas 3135 genes displayed decreased expression in purple petals relative to white petals. This gene expression divergence highlights potential regulatory and metabolic pathways underlying the distinct petal coloration. The anthocyanin synthesis pathway is co-regulated by numerous enzymes and genes, among which various enzymes play crucial roles. Multiple enzymes are key in regulating the total anthocyanin content, such as cinnamate 4-monooxygenase (C4H), 4-coumarate-CoA ligase (4CL), chalcone isomerase (CHI), flavanone 3-hydroxylase (F3H), dihydroflavonol 4-reductase (DFR), anthocyanidin synthase (ANS), flavonoid 3-*O*-glucosyltransferase (UF3GT), and flavonoid 3,5-*O*-glucosyltransferase (UF35GT). The enzymes at the branch points, F3′H and F3′5′H, dictate the overall anthocyanin concentration and the relative proportions of the primary components. A total of 10 PAL, 2 CHS, 1 ANR, 1 4CL, 1 DFR, 1 F3′H, and 1 FLS structural genes were screened out. Their expression levels in purple flowers were higher than those in white flowers, which may contribute to the anthocyanin accumulation in purple flowers.

In addition to structural genes, researchers have identified many transcription factors (TFs) that regulate the activity of structural genes, hence affecting anthocyanin levels and floral pigmentation [34,35,36,37,38,39]. The synthesis of anthocyanins is precisely regulated by TFs during plant development. MYB, bHLH (basic helix-loop-helix), and WD40 (WD40-repeat) TFs, as the MYB-bHLH-WD40 (MBW) complex, jointly control the expression of anthocyanin biosynthesis genes [40,41,42]. Studies have shown that HabHLH1 and HaMYBA synergistically regulate the increased expression of dihydroflavonol 4-4-reductase (DFR), leading to anthocyanin accumulation. In Jiang’s research, HaMYBA was shown to interact with HabHLH1 and enhance anthocyanin production, whereas HaMYBF stimulates the flavonol pathway but diminishes anthocyanin accumulation [43,44]. Research reveals that MYB, bHLH, WD40, and WRKY are significant transcription factors associated with floral color, playing a role in the regulation of anthocyanin production and metabolic pathways. In this paper, for these four TF families, some TFs with an absolute log2FoldChange value greater than 2 were screened, including 15 WRKY, 5 bHLH, and 8 MYB.

**Figure 5:**
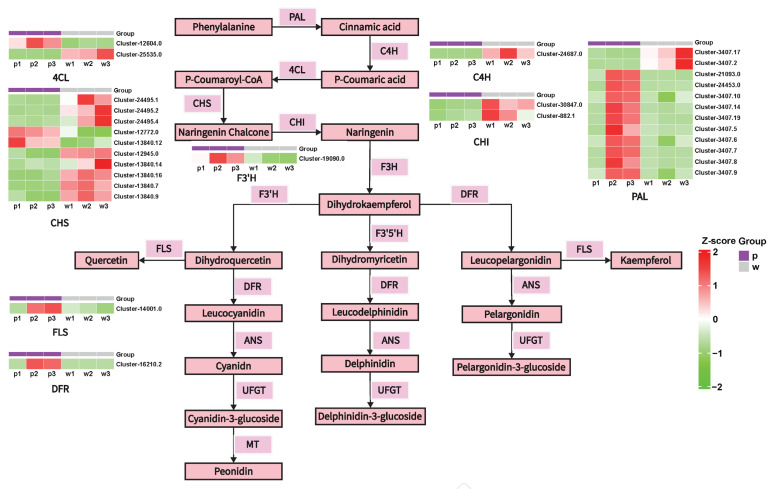
Visualization of the Flavonoid Metabolic Pathway Involving Key Enzymes and the Heatmap of Metabolite Contents.

We mapped the synthesis pathway of anthocyanins (Figure 5 and Figure S4). Based on previous studies, delphinidin is usually closely associated with a blue or purple appearance [45,46,47]. The purple petals in our experiment further validate this argument. The content of delphinidin in the metabolome is several times higher than that of other anthocyanins. Therefore, we focused on the pathway where dihydrokaempferol is synthesized into dihydromyricetin under the action of F3′5′H. Notably, as an important structural gene, DFR (dihydroflavonol 4-reductase) has an expression level in purple flowers that is far higher than that in white flowers. It is the gene with the most significant difference between the two-colored petals. Thus, we consider DFR to be a crucial gene for the synthesis of purple anthocyanins. This study has unveiled the potential formation mechanisms of anthocyanins in the two *Iris dichotoma* varieties, providing a theoretical basis for flower breeding.

## 4. Methods

### 4.1. Plant Materials

Purple and white iris blooms were cultivated at the nursery of the Institute of Grassland Research, Chinese Academy of Agricultural Sciences, located in Hohhot, Inner Mongolia (111.8° E, 40.6′ N, China). In June 2024, fresh petals were collected from flower bud samples. Plant specimens underwent cryopreservation in liquid nitrogen followed by maintenance at −80 °C prior to experimental application. The experimental design incorporated triplicate biological trials, with each trial involving petal tissues pooled from three distinct specimens. Quantitative assessment of floral pigmentation characteristics was conducted through spectrophotometric evaluation at peak floral maturity.

### 4.2. Extraction, Separation, Identification, and Quantification of Anthocyanins

On the AB SCIEX QTRAP 6500 LC-MS/MS platform, MetWare Biotechnology Company (Wuhan, China; http://www.metware.cn/) conducted the detection of anthocyanin accumulations. Initially, biological samples underwent vacuum freeze-drying to eliminate moisture and preclude component degradation. Subsequently, they were comminuted into fine particles via ball milling at a frequency of 30 Hz for 90 s, aiming to optimize their interfacial interaction with the extraction medium. Then, 50 mg of the resulting powder was dissolved in 500 μL of a 50% methanol-water solution containing 0.1% hydrochloric acid. The mixture was vortexed and sonicated for 5 min each to enhance component dissolution. The supernatants from the two extraction procedures were combined and filtered through a 0.22 μm microporous membrane. The filtrate was then stored in an injection vial for subsequent LC-MS/MS analysis. The analytical system employed an ExionLC™ AD ultra-high-performance liquid chromatography system integrated with a QTRAP^®^ 6500+ tandem mass spectrometer. For liquid chromatography, an ACQUITY BEH C18 column (1.7 µm, 2.1 mm × 100 mm) was utilized. The mobile phase consisted of mobile phase A, which was ultrapure water with 0.5% formic acid, and mobile phase B, which was methanol with 0.5% formic acid. The elution gradient was configured as follows: The proportion of phase B increased from 5% at 0 min to 50% at 6 min, further escalated to 95% at 12 min (held for 2 min), decreased to 5% at 14 min, and was then equilibrated for 2 min. The flow rate was maintained at 0.35 mL/min, the column temperature was set at 40 °C, and the injection volume was 2 μL.

Regarding mass spectrometry, an electrospray ionization source (ESI) was operated in the positive-ion mode at a temperature of 550 °C, a voltage of 5500 V, and a curtain gas pressure of 35 psi. Ion pairs were analyzed and identified based on optimized declustering potentials and collision energies. The content of anthocyanins was determined using the scheduled multiple reaction monitoring (MRM) mode. Data acquisition was executed via the Analyst 1.6.3 software, and quantitative analysis of metabolites was performed using the Multiquant 3.0.3 software.

### 4.3. RNA-Seq

In strict accordance with the operation instructions, impurities and DNA contamination were removed through multiple washing and centrifugation steps. The RNA concentration was measured with high precision using a Qubit 4.0 fluorometer and an MD microplate reader. The RNA integrity was accurately detected using a Qsep400 bio-analyzer to ensure that the OD260/OD280 ratio was between 1.8 and 2.0 and the RNA was in good integrity. After the RNA samples were verified to be error-free, mRNA was isolated and cDNA was synthesized. Double-stranded cDNA was ligated to adapters, followed by screening, amplification, and purification to generate a cDNA library. After the library construction was completed, preliminary quantification was first performed using a Qubit, and then the inserted fragments of the library were detected using an Agilent 2100 (Agilent Technologies, Baden-Württemberg, Germany) to ensure the quality of the library. Sequencing was carried out using the Illumina Novaseq6000 system at Metware Biotechnology (Wuhan, China).

For samples without a reference genome, after obtaining clean reads, Trinity [48] was employed for splicing. To obtain high-quality readings, the raw data were filtered. The transcript sequences of the species were obtained through Trinity splicing, and then Unigene sequences were obtained using the corset. The high-quality reads were aligned with the de-transcribed transcripts to calculate the gene expression levels. Finally, the differentially expressed genes in different grouped samples were calculated, and the differential genes were annotated and subjected to enrichment analysis.

### 4.4. Weighted Gene Co-Expression Network Analysis

The weighted gene co-expression network analysis (WGCNA) R package (4.3.0) was utilized for co-expression analysis. Initially, an unsigned topological overlap measure was employed to construct the network and identify modules within the dataset. The parameters included a minimum module size of 30, a merge cut height of 0.25, and the soft-threshold power was enabled. To rank each gene within the module, the intramodular connectivity of each gene was calculated, and the top 15 genes in the hub were selected. The Pearson correlation coefficient (PCC) was computed to assess the association at the module stage. The resulting modules were visualized in Cytoscape v3.7.1 [49].

### 4.5. qRT-PCR

We additionally confirmed the expression patterns of differentially expressed genes (DEGs) identified through RNA sequencing using quantitative real-time PCR (qRT-PCR). We randomly chose 15 differentially expressed genes for analysis using gene-specific primers (Figure S1). The qRT-PCR study verified that the chosen genes exhibited differential expression in purple and white lines.

## 5. Conclusions

In this investigation, purple-flowered and white-flowered Iris specimens were employed as experimental materials. Through the comprehensive integration of phenotypic analysis, transcriptomic profiling, and metabolomic assays, an in-depth exploration was conducted into the latent mechanisms governing the formation of Iris flower coloration. Metabolomic analysis indicated that the abundances of several anthocyanin derivatives within purple petals, including delphinidin, petunidin, malvidin, peonidin, and proanthocyanidins, were markedly elevated compared to those in white petals. Among these, delphinidin exhibited the highest concentration, thereby making the most substantial contribution to the manifestation of the purple hue. Transcriptomic analysis identified ten phenylalanine ammonia-lyase (PAL) genes, two chalcone synthase (CHS) genes, one anthocyanidin reductase (ANR) gene, one 4-coumarate-CoA ligase (4CL) gene, one dihydroflavonol 4-reductase (DFR) gene, one flavanone 3′-hydroxylase (F3′H) gene, and one flavonol synthase (FLS) gene. These twenty structural genes, in conjunction with numerous transcription factors (TFs), were intricately associated with the development of purple flower color. Collectively, this study has illuminated the functions of crucial anthocyanins and related genes in the formation of Iris flower color, furnishing a theoretical foundation for subsequent investigations.

## Data Availability

All data are open and available. The raw data are available in the NCBI database (BioProject ID PRJNA1244055) (https://www.ncbi.nlm.nih.gov/sra?term=URL&cmd=DetailsSearch&log$=activity) (accessed on 30 March 2025).

## Supplementary Materials

The following supporting information can be downloaded at: https://www.mdpi.com/article/10.3390/plants14091387/s1, Figure S1: Verification of 15 DEGs by qRT-PCR; Figure S2: DAE Heatmap of Anthocyanins in Purple and White Petals (P and W groups); Figure S3: Relationship Map of Key Transcription Factors (WRKY, bHLH, MYB) and Structural Genes in Anthocyanin Biosynthesis; Figure S4: Visualization of the Flavonoid Metabolic Pathway Involving Key Enzymes and the Heatmap of Metabolite Contents.
