# Supplementary figures and images for: In-Depth Exploration of the Coloration Mechanism of Iris dichotoma Pall. via Transcriptomic and Metabolomic Analyses
Source: Plants (Basel). 2025 May 4;14(9):1387. doi: 10.3390/plants14091387 (PMC12073270; doi:10.3390/plants14091387)

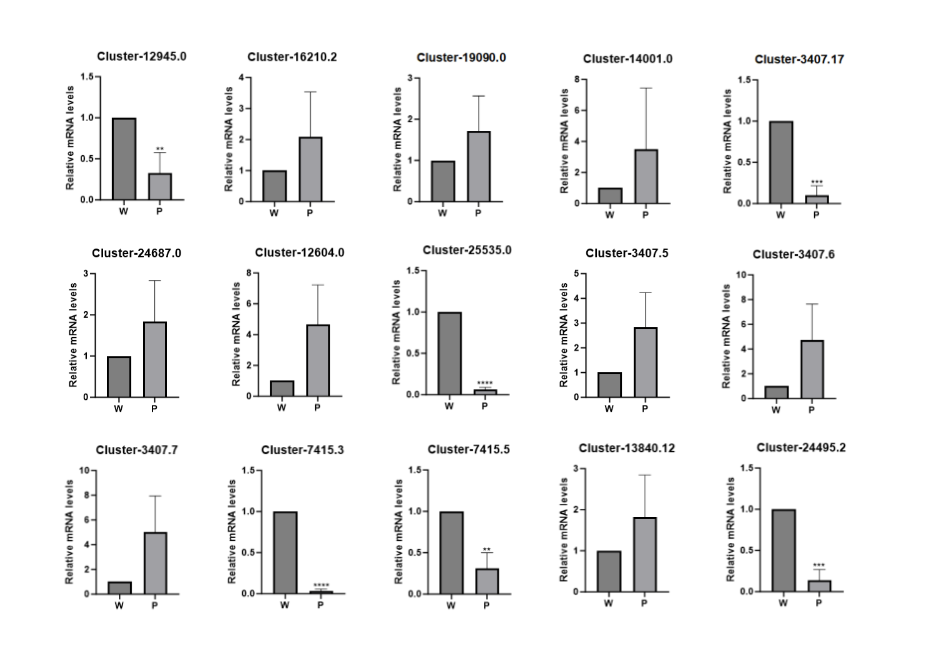

Supplement: Supplementary file 1 [file plants-14-01387-s001.zip › plants-3605097-supplementary/Figure S1. Verification of 15 DEGs by qRT-PCR; Figure .png]

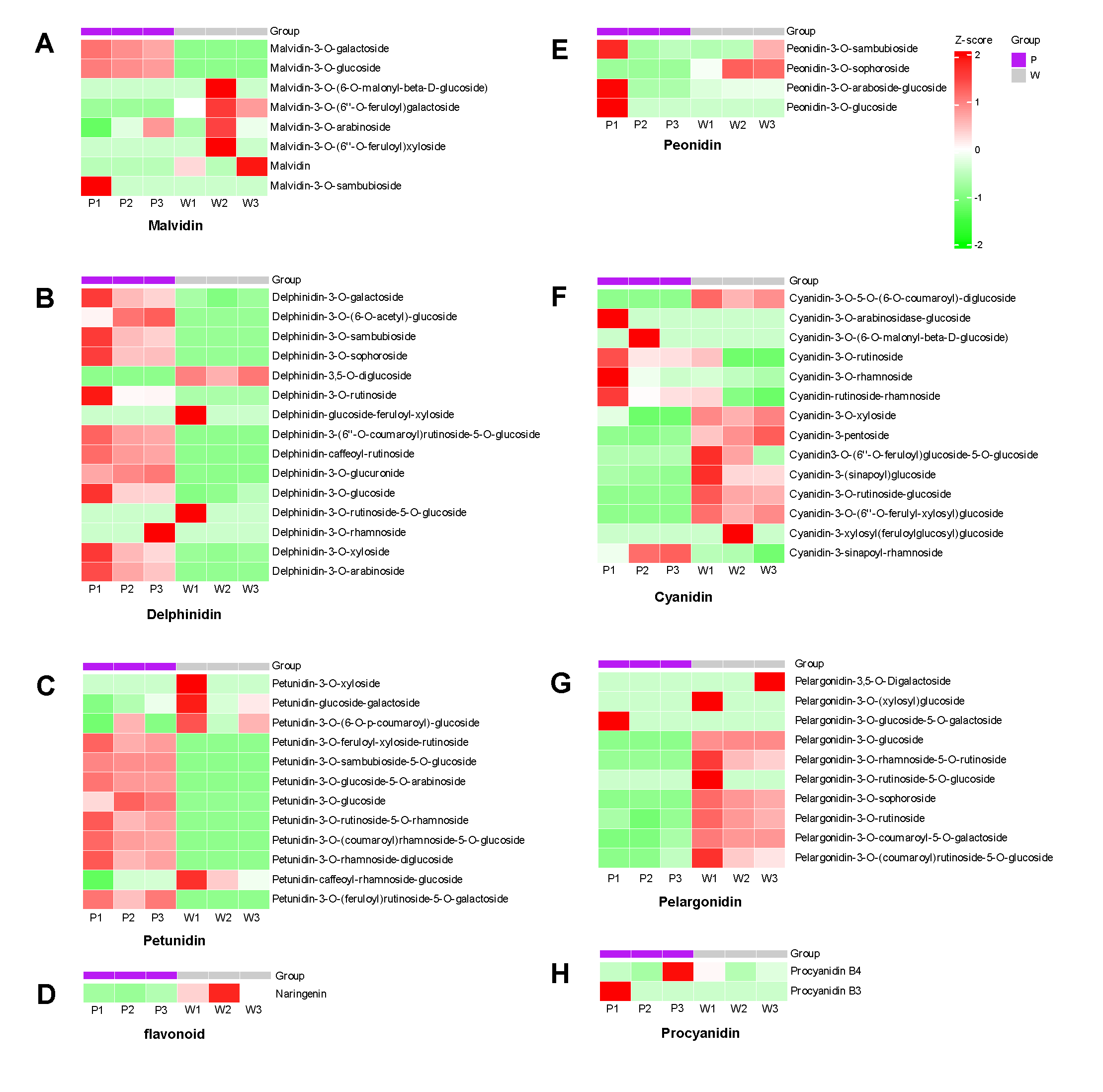

Supplement: Supplementary file 1 [file plants-14-01387-s001.zip › plants-3605097-supplementary/Figure S2. DAE Heatmap of Anthocyanins in Purple and White Petals (P and W groups).jpg]

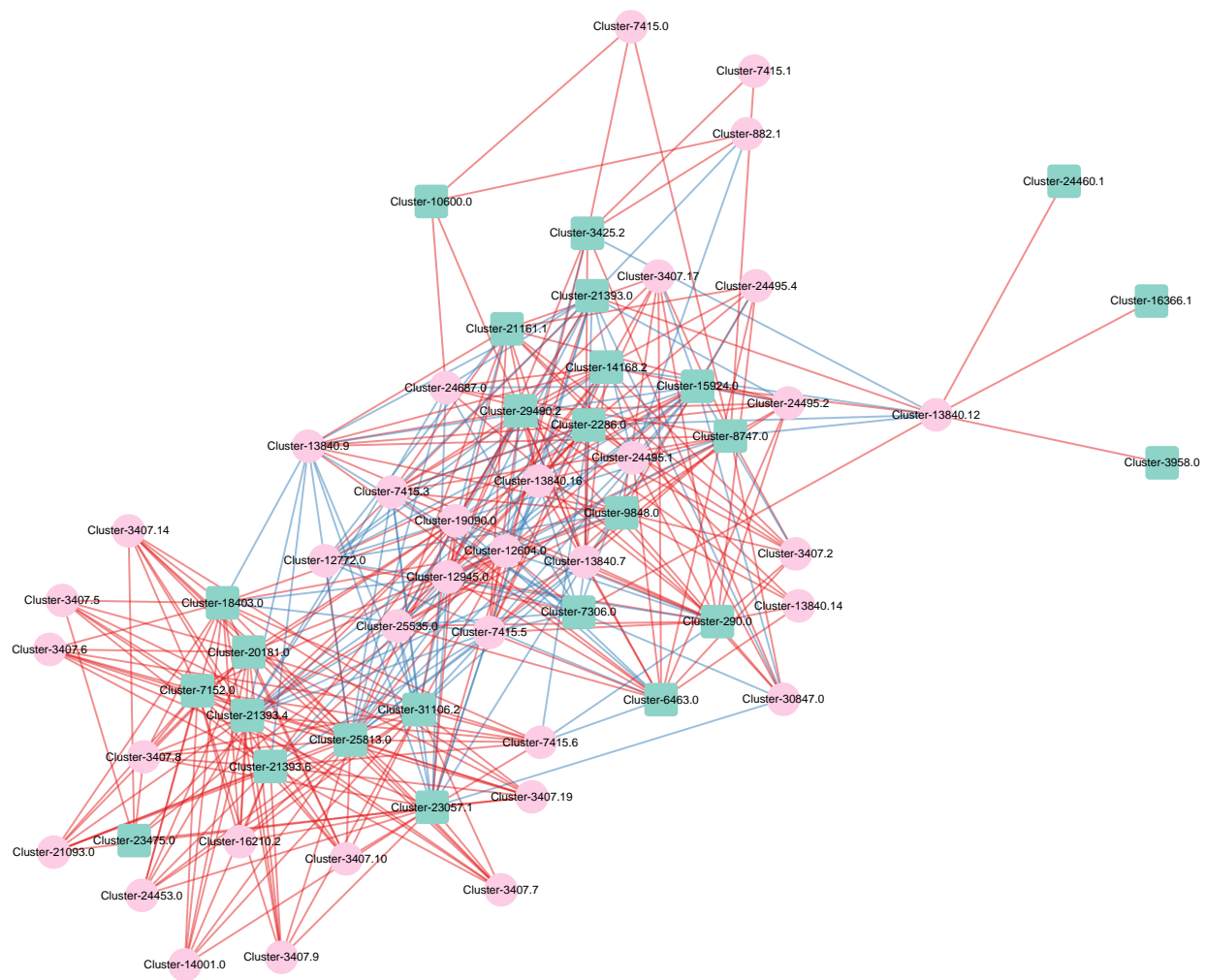

Supplement: Supplementary file 1 [file plants-14-01387-s001.zip › plants-3605097-supplementary/Figure S3. Relationship Map of Key Transcription Factors (WRKY, bHLH, MYB) and Structural Genes in Anthocyanin Biosynthesis.pdf]

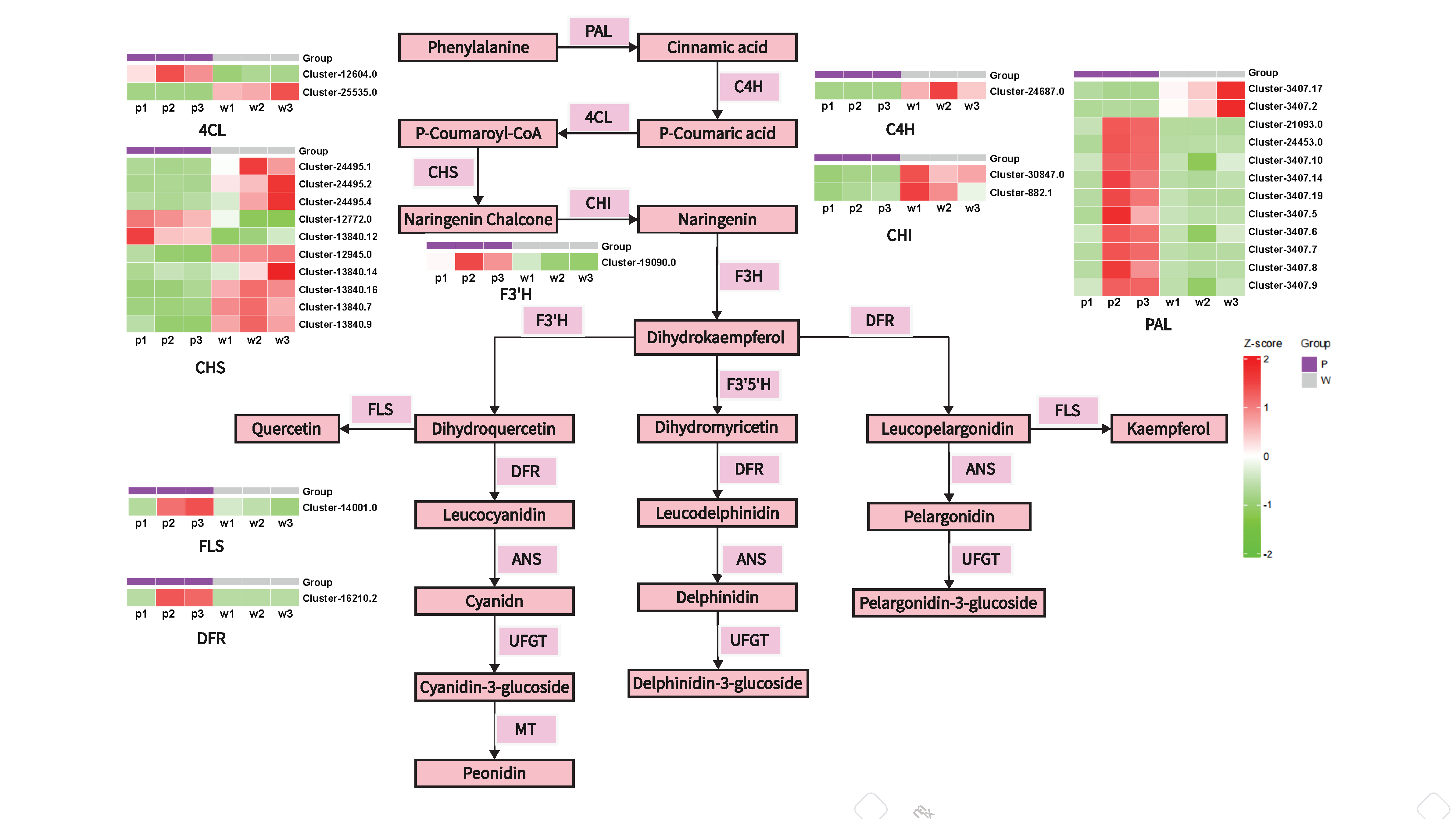

Supplement: Supplementary file 1 [file plants-14-01387-s001.zip › plants-3605097-supplementary/Figure S4. Visualization of the Flavonoid Metabolic Pathway Involving Key Enzymes and the Heatmap of Metabolite Contents.png]
